# Visual motherese? Signal-to-noise ratios in toddler-directed television

**DOI:** 10.1111/desc.12156

**Published:** 2014-04-07

**Authors:** Sam V Wass, Tim J Smith

**Affiliations:** 1 Medical Research Council Cognition and Brain Sciences UnitCambridge, UK; 2 School of Psychological Sciences, Birkbeck College, University of LondonUK

## Abstract

Younger brains are noisier information processing systems; this means that information for younger individuals has to allow clearer differentiation between those aspects that are required for the processing task in hand (the ‘signal’) and those that are not (the ‘noise’). We compared toddler-directed and adult-directed TV programmes (TotTV/ATV). We examined how low-level visual features (that previous research has suggested influence gaze allocation) relate to semantic information, namely the location of the character speaking in each frame. We show that this relationship differs between TotTV and ATV. First, we conducted Receiver Operator Characteristics analyses and found that feature congestion predicted speaking character location in TotTV but not ATV. Second, we used multiple analytical strategies to show that luminance differentials (flicker) predict face location more strongly in TotTV than ATV. Our results suggest that TotTV designers have intuited techniques for controlling toddler attention using low-level visual cues. The implications of these findings for structuring childhood learning experiences away from a screen are discussed.

## Introduction

### Signal-to-noise and the developing brain

Since William James first observed that an infant experiences the world as a ‘blooming, buzzing confusion’ (James, 1890, p. 487) convergent research has supported the idea that younger brains are relatively noisier information processing systems: local connectivity is higher (Kelly, Di Martino, Uddin, Shehzad, Gee, Reiss, Margulies, Castellanos & Milham, 2009), long-distance connectivity is lower (Fair, Cohen, Dosenbach, Church, Miezin, Barch, Raichle, Petersen & Schlaggar, 2008), cortical functional activation patterns are relatively less localized and specialized (Johnson, 2010) and excitatory/inhibitory balances are more unstable (Froemke and Jones, 2011).

Evidence supporting James' intuition has also been observed in behavioural research. Adults can judge the identity and order of changing images presented at a rate of up to 10 Hz; for 15-month-old infants the equivalent maximum is 1 Hz (Farzin, Rivera & Whitney, 2012). In language acquisition, the influence of lexical neighbor density on word learning declines with increasing age (Storkel, 2009). In cognitive control, distractor–target similarity affects performance on Stroop-like tasks more in younger than in older individuals (Montgomery & Koeltzow, 2010). On delayed response tasks, research with infants suggests that more salient visual cues can improve response accuracy following longer time delays (Clearfield, Dineva, Smith, Diedrich & Thelen, 2009). Research suggests that in order to achieve equivalent comprehensibility on a learning task, information presented to younger individuals has to be structured with clearer differentiation between those aspects of the stimulus that are required for the learning task in hand (the ‘signal’) from those that are not (the ‘noise’) (Rost & McMurray, 2010; Swingley & Aslin, 2007; Thiessen, 2011; Yu & Smith, 2012).

One particularly well-known example of this is infant-directed speech (IDS, also known as motherese), which contains exaggerated pitch, elongated words and expanded vowel space with stretched formant frequencies (Ferguson, 1964). Previous authors have suggested that these changes entail an exaggeration of the critical features that distinguish one phoneme from others (Pierrehumbert, 2003; Zhang, Koerner, Miller, Grice-Patil, Svec, Akbari, Tusler & Carney, 2011). Infants learn better from IDS than they do from adult-directed speech (ADS) (Swingley, 2009), and IDS evokes greater attention-related electrophysiological responses than ADS (Zhang *et al*., 2011).

### Toddler-directed screen media

The average American child aged 0–6 years spends 96 minutes per day watching screen media (including TV, DVDs, and video games), compared to only 49 minutes reading or being read to (Rideout & Hamel, 2006; Wartella, Richert & Robb, 2010). Although exposure to programmes such as *Sesame Street* has been associated with improved school readiness and academic performance (Anderson & Hanson, 2010), other research has identified a ‘video deficit effect’ – that infants and young children find it hard to transfer learning from TV to real-life situations (Barr, 2010).

Designers of children's television face a similar problem to that of a caregiver attempting to verbally communicate to their child: how do you best structure the audiovisual content to facilitate comprehension? A survey of CBeebies, the British Broadcasting Corporation (BBC) channel aimed at the 0–6-years age range, reveals differences in style and content between toddler-directed TV (TotTV) and adult-directed TV (ATV).^1^ Of the top-10-rated programmes on CBeebies, five are animated (computer-generated animation (CGA) and Cel), and two of the remaining five feature exclusively puppet-like faces;^1^ in contrast, all of the top-10-rated BBC programmes for adults are live action with humans. Their content also differs on other parameters such as length and narrative complexity (Gunter & McAleer, 1997). Cy Schneider, a founding producer at Nickelodeon, states that preschoolers ‘are not yet capable of putting the parts together and only retain fragments of any particular message. Their comprehension increases when the visual part of the message is very clearly defined. They also react better to passive, quiet television programming that is organized in short bursts’ (Schneider, 1987, p. 85). He advises using large changes in colour or scene to indicate structure and clear contrast between foreground and background objects to denote relevance.

In cognitive science, research has identified a number of ways in which the low-level visual features of a stimulus can influence gaze behaviour (Itti & Koch, 2001; Itti & Baldi, 2009). High contrasts in ‘first-order’ static stimulus features such as luminance and colour intensity have been shown in classic attentional cueing paradigms to act as exogenous attentional cues (see Wolfe & Horowitz, 2004, for review). However, ‘second-order’ static features such as feature congestion (defined as a combination of differentials of luminance, colour and edge orientation) are thought to play a larger role in influencing gaze allocation during dynamic scene viewing (Rosenholtz, Li & Nakano, 2007). The strongest low-level cue to gaze allocation, however, is thought to be movement (Mital, Smith, Hill & Henderson, 2010). This is often approximated by calculating flicker, i.e. luminance differentials across frames. Low-level salience cues are thought to operate during dynamic scene viewing according to a ‘winner-takes-all’ model, possibly in combination with other factors such as Inhibition of Return, although this remains controversial (Carmi & Itti, 2006; Itti & Baldi, 2009; Itti & Koch, 2001; Smith & Henderson, 2011; Tatler, Hayhoe, Land & Ballard, 2011).

Research with infants and children has suggested that low-level features influence gaze behaviour relatively more strongly in very young individuals than they do in adults (Frank, Vul & Johnson, 2009; Kirkorian, Anderson & Keen, 2011; Valkenburg & Vroone, 2004). Frank and colleagues compared the role that low-level features and semantic factors (i.e. whether or not a given pixel is part of a character face) play in predicting gaze location in 3-, 6- and 9-month-old infants, as well as in adults. They found that the relative role that low-level factors play in predicting gaze location declines with increasing age (Frank *et al*., 2009; see also Smith, Dekker, Mital & Karmiloff-Smith, under review; Gola & Calvert, 2011). In young infants, salience cues play a greater role than content (face/non-face) in guiding gaze allocation; in older infants and adults, low-level features continue to guide gaze allocation but with increasing age semantic factors additionally play a role (cf. Smith & Mital, 2013).

To our knowledge, no research hitherto has directly investigated how TotTV and ATV differ in terms of computationally quantified low-level features (although see Gola & Calvert, 2011; Goodrich, Pempek & Calvert, 2009; Calvert, Huston, Watkins & Wright, 1982, for hand-coded equivalents). Furthermore, no research has investigated how low-level features are used to guide attention to the semantically important aspects of the scene (‘the signal’) relative to the semantically unimportant aspects (‘the noise’), and whether this differs between TotTV and ATV. In the present study we wished to examine these questions using corpus analyses (cf. Cutting, DeLong & Nothelfer, 2010). Since we were analysing TV dramas we concentrated on one parameter of semantic content, namely the location of the face of the character speaking in each frame; this was identified via hand-coding. We then calculated low-level features frame by frame: we calculated two first-order static features (luminance, colour intensity), a second-order static feature (feature congestion) and a first-order dynamic feature (flicker, which approximates motion) (Mital *et al*., 2010). We then analysed the role that low-level stimulus features play in predicting the location of the speaking character. We hypothesized that TotTV would show a higher proportion of low-level attention cues around the speaking character (‘the signal’), with a lower proportion of exogenous attention cues in the rest of the frame (‘the noise’). We compared the most popular TV dramas on CBeebies, the BBC channel aimed at 0–6-year-old children in the UK, with the most popular TV dramas on BBC1, the main adult BBC channel.

## Methods

### Stimuli

**Figure 1:**
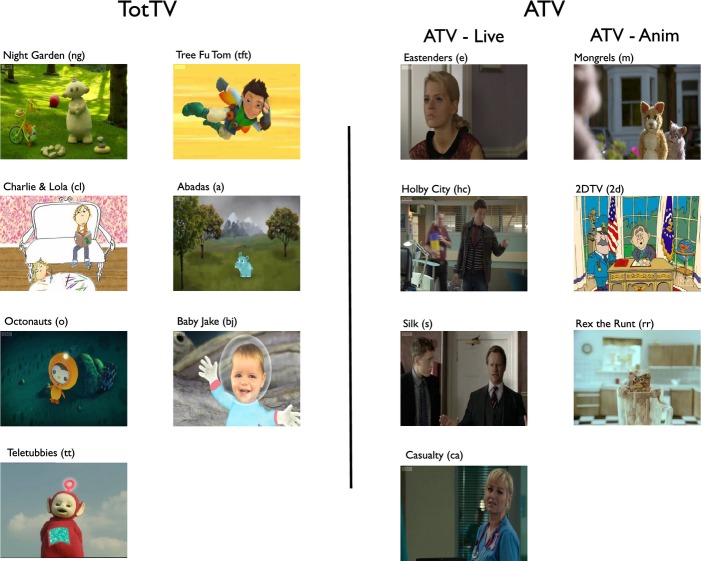
The clips included in our sample. Our stimuli were classified into the following categories: Cel (hand-drawn animation), CGA (computer-generated animation) or LA (live action). TotTV: Night Garden (LA); Charlie & Lola (mixed Cel/LA); Octonauts (CGA); Teletubbies (LA); Tree Fu Tom (CGA); Abadas (Cel/LA); Baby Jake (CGA/LA). ATV-Live: Eastenders (LA); Holby City (LA); Silk (LA); Casualty (LA). ATV-Anim: Mongrels (LA); 2DTV (Cel); Rex the Runt (CGA).

The selection of television programmes was conducted based on viewing figures from the British Audience Research Bureau ratings for the week ending 27 May 2012^2^ for the CBeebies channel^3^ and from BBC1. Stimuli were classified into Live Action (LA), Cel (hand-drawn) animation and computer-generated animation (CGA) (see Figure 1). All of the popular TotTV excerpts contained animation or live action featuring puppets, whereas all of the popular ATV excerpts were live action with human actors. We therefore also included a selection of adult-directed animations; since none of the top 30 programmes on BBC1 were animated, these were selected from BBC3 and ITV (see Figure 1). Selection of programmes for analysis was conducted blind; the segments analysed are included in the Supplementary Materials.

For each clip the last frame of the title sequence was identified. A frame-by-frame decomposition of the 5 minutes after this point was performed, splitting the video at 25 frames per second into a 400 × 300 pixel format using Psychtoolbox and Matlab, on which all subsequent analyses were based. In total 105,000 frames (7500 per sample) were included in our analysis (see Cutting *et al*., 2010, for a similar approach).

### Coding of cuts

First we hand-identified cuts in order to exclude them from our movement-based analyses; dissolves were included. In order to confirm inter-rater reliability, a second coder who was naive to the purpose of the study double-coded 20% of the data. Cohen's kappa was found to be 0.98.

### LAB decomposition

**Figure 2:**
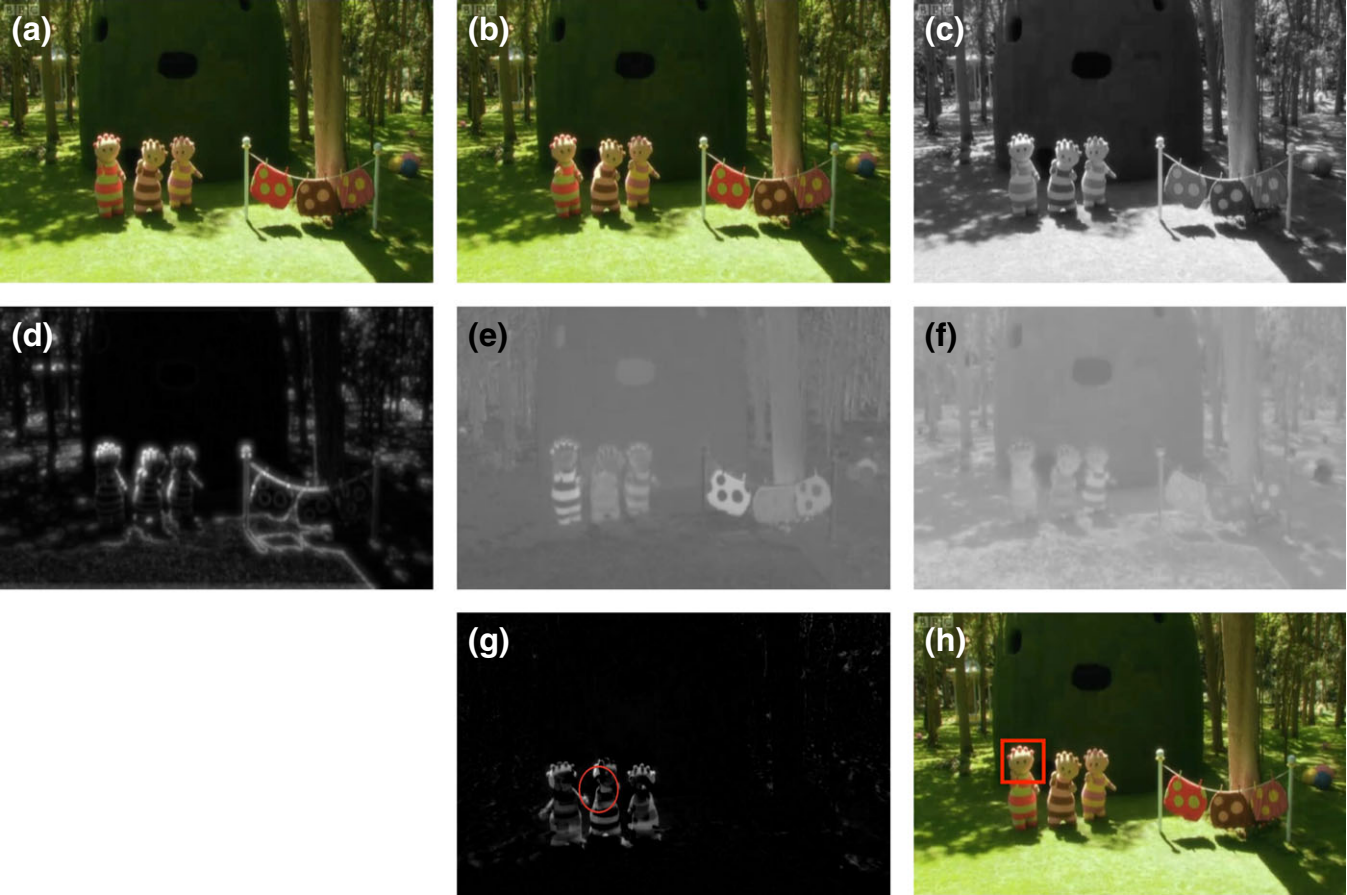
A demonstration of the transformations we applied, that are described in the main text. (a) and (b) show two consecutive frames from In the Night Garden by the BBC; (c) shows the luminance of frame a; (d) shows the feature congestion; (e) shows the red–green colour spectrum; (f) shows the blue–yellow colour spectrum; (g) shows flicker, the difference in luminance between frames a and b. The red circle shows the centre and variance of the change in flicker (see Methods). (h) shows the position of the speaking character, as identified via hand-coding.

A number of researchers have studied how luminance and colour contribute to visual saliency (Parkhurst & Niebur, 2003). We separated luminance and colour using the 1976 CIE L*a*b* (CIELAB) colour-space convention (see Figure 2 and Supplementary Methods (SM)).

### Feature congestion

Feature congestion is local variability across different image features such as colour, orientation and luminance (Rosenholtz *et al*., 2007). It is comparable to the more widely used salience measure (Itti & Koch, 2001; see Figure 2 and SM)

### Flicker decomposition

Frame-to-frame changes in luminance, or flicker, have been shown to attract attention during free-viewing (Theeuwes, 1995). To calculate flicker we converted each frame into the CIELAB colour-space to derive the luminance and then computed the absolute difference in luminance between the current and previous frames (Mital *et al*., 2010). In addition, we also calculated the centre and spatial dispersal of the flicker on a frame-by-frame basis (see Figure 2 and SM).

### Speaking character coding

Speaking character coding was conducted by hand to identify the face of the character speaking in each frame (see Figure 2). Coding was performed in 1-second segments using specially written Matlab scripts. These played each clip for a second and then froze; the coder drew a rectangle around the face of the last character speaking or being addressed by a narrator. If no characters had spoken in that second then no speaking character was marked. Segments in which the face of the speaking character was not visible (e.g. shots of the back of the head) were excluded. The mean (*SD*) number of codable intervals per sample was TotTV: 137 (56); ATV-Live: 168 (32); ATV-Anim: 186 (27) (in each case out of a maximum of 300). In order to assess inter-rater reliability, a second coder who was naive to predicted outcomes double-coded a 20% section of the data. Cohen's kappa was calculated based on the probability that each pixel was within the speaking character frame; this was 0.96.

### ROC analysis

In order to assess the selection of visual features we employed a signal detection framework based on the Receiver Operator Characteristic (ROC) (Le Meur, Le Callet & Barba, 2007). This analyses the degree to which each feature predicts the location of the face relative to the location of a randomly sampled baseline location, frame by frame (see SM).

## Results

Our analysis is in four parts. First we present descriptive analyses for the different categories of TV clips: (1) results from hand-coding of cut duration and percentage of screen occupied by speaking character and (2) results from feature decomposition of analyses of the total amounts of each feature parameter contained across the whole sample. Second we present our signal-to-noise analyses in two stages: (3) static features and (4) dynamic features.

### Analysis strategy

Results are presented grouped by category: toddler-directed TV (TotTV) and adult TV (ATV) (see Table 1). Adult has been subdivided into adult – animations (ATV-Anim) and adult – live action (ATV-Live). All analyses are reported as independent samples *t*-tests; two-tailed *p*-values have been reported. Two analyses were conducted for all comparisons: first we compared TotTV and ATV; second, in order to evaluate the degree to which our findings are attributable to the difference between animation and live action, we also compared ATV-Live and ATV-Anim.

**Table 1:** Results of main analyses. a - indicates results are x 10∧3

	TotTV	ATV			Sig.
			ATV-Live	ATV-Anim	
**1) Hand-coding**					
Average shot length (secs)	7.26 (2.1)	3.3 (1.2)	2.9 (0.6)	4.0 (1.9)	TotTV>ATV
Average proportion of screen occupied by speaking character	0.075 (0.04)	0.13 (0.03)	0.14 (0.03)	0.13 (0.05)	TotTV<ATV
**2) Feature decomposition**					
Total luminance	134 (10)	80 (4)	80 (4)	131 (21)	TotTV>ATV; ATV-Anim>ATV-Live
Total feature congestion	4.0 (1.3)	3.3 (2.0)	2.4 (0.4)	4.5 (2.8)	
Total blue-yellow intensity	20 (11)	9.2 (4.5)	6.3 (2.4)	13 (3.8)	TotTV>ATV; ATV-Anim>ATV-Live
Total red-green intensity	12 (4)	3.7 (1)	3.2 (1.1)	4.3 (1.6)	TotTV>ATV
Total flicker	2.2 (0.6)	2.7 (0.9)	2.8 (0.6)	2.5 (1.3)	
**3) Signal-to-noise: static features**					
AUC (luminance)	0.53 (0.06)	0.52 (0.05)	0.50 (0.05)	0.55 (0.04)	
AUC (feature congestion)	0.59 (0.05)	0.52 (0.03)	0.50 (0.008)	0.55 (0.03)	TotTV>ATV; ATV-Anim>ATV-Live
AUC (blue-yel intensity)	0.50 (0.01)	0.50 (0.007)	0.50 (0.004)	0.50 (0.008)	
AUC (red-green intensity)	0.50 (0.01)	0.50 (0.10)	0.50 (0.003)	0.50 (0.01)	
**4) Signal-to-noise: dynamic features**					
Average dispersal in flicker	1.89 (0.06)	2.17 (0.06)	2.2 (0.04)	2.1 (0.1)	TotTV<ATV
Distance between centre of flicker and centre of speaking character face ^a^	28 (2.3)	37 (8.0)	42 (2.6)	29 (1.6)	TotTV<ATV; ATV-Anim<ATV-Live
AUC (flicker)	0.54 (0.02)	0.52 (0.03)	0.50 (0.008)	0.55 (0.03)	TotTV>ATV-Live

#### Hand-coding

##### Shot length (see Figure 3a– 3b)

In total, 871 cuts were identified across the 14 excerpts. Mean cut duration was longer in TotTV than ATV (*t*(1,12) = 2.88, *p* < .05). The ATV-Live vs. ATV-Anim comparison was not significant (*t*(1,5) = 1.3, *p* = .25).

**Figure 3:**
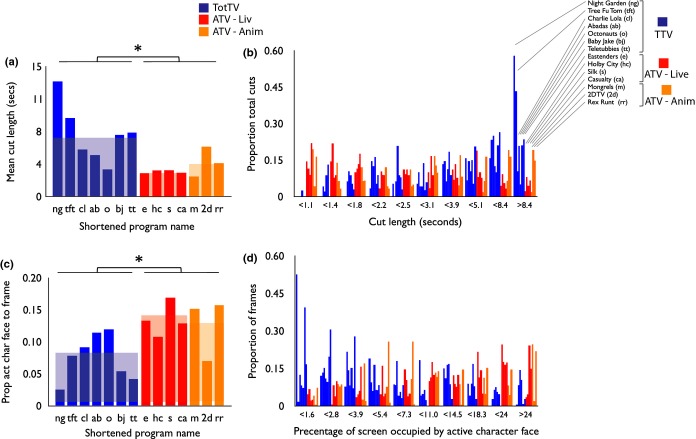
(a) Average cut length. Each bar shows the average cut length for that sample. The shortened name for each programme is given on the x-axis (see Figure 1). The faded background colour indicates the mean for each category; (b) histograms of cut length, divided by sample. The length of cut (in seconds) is shown on the x-axis. The proportion of cuts within that bin have been drawn on the y-axis; (c) proportion of screen area occupied by speaking character face; (d) histograms showing the percentage of screen occupied by the speaking character face for each programme. The clips have been ordered as in Figure b). Stars indicate the significance of the group comparisons reported in the text. *p < .05.

##### Proportion of screen occupied by speaking character (see Figure 3c– 3d)

Percentage of screen occupied by the speaking character across all frames was significantly lower in TotTV than ATV: (*t*(1,12) = 3.0, *p* < .05). The ATV-Live vs. ATV-Anim comparison was not significant.

Calculations were also conducted to assess the relationship between shot length and proportion of screen occupied by the speaking character (see Figure S1). Across all three categories negative correlations were observed, suggesting that longer cuts are associated with the speaking character occupying a smaller proportion of the screen. Pearson product-moment correlations were calculated to assess the strength of this relationship independently in the three categories and revealed that the relationship was stronger in TotTV (*r*(1,248) = −.32, *p* < .001) and ATV-Anim (*r*(1,206) = −.15, *p* < .05) than ATV-Live (*r*(1,305) = −.10, *p* = .07) (see Figure S1).

#### Feature decomposition

**Figure 4:**
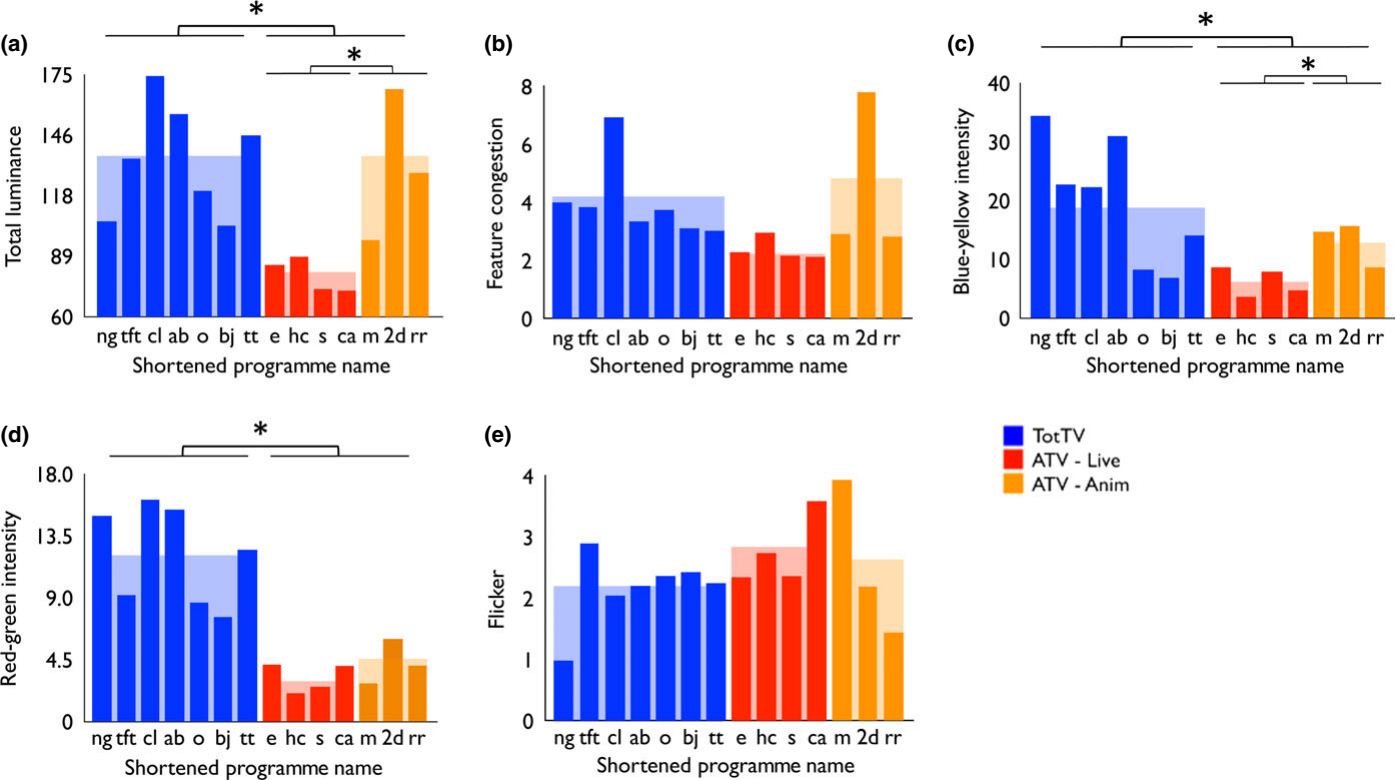
The total mean values of each feature parameter (luminance, colour intensity, feature congestion and flicker), irrespective of whether where that content is located in the frame. (a) Total luminance; (b) feature congestion; (c) blue–yellow intensity; (d) red–green intensity; (e) flicker. Stars indicate the significance of the group comparisons reported in the text. *p < .05.

First we analysed the mean values of each feature parameter (luminance, colour intensity, feature congestion and flicker) across the entire segment, irrespective of whether that content is located around the face of the speaking character or elsewhere in the frame ( Figures 4a– 4d).

##### Mean total luminance

This was higher in TotTV than ATV (*t*(1,9) = 3.9, *p* < .01). Within ATV, luminance was higher in ATV-Anim than ATV-Live (*t*(1,5) = 2.85, *p* < .05).

##### Feature congestion

Comparison of TotTV and ATV was not significant (*t*(1,12) = .78, *p* = .46). Comparison of ATV-Live and ATV-Anim 4.5 (2.8) was also not significant (*t*(1,5) = 1.5, *p* = .19).

##### Colour intensity

Blue–yellow intensity was higher in TotTV than ATV (*t*(1,12) = 2.5, *p* < .05). With the ATV group, it was higher in ATV-Anim than ATV-Live (*t*(1,5) = 2.9, *p* < .05). Red–green intensity was higher in TotTV than ATV (*t*(1,12) = 5.8, *p* < .001). The comparison of ATV-Live and ATV-Anim was not significant (*t*(1,5) = 1.1, *p* = .33). The comparison of TotTV and ATV-Anim was not significant for blue–yellow (*t*(1,8) = 1.1, *p* = .32) but was for red–green (*t*(1,8) = 3.5, *p* < .01).

##### Flicker

This showed no significant difference between TotTV and ATV (*t*(1,12) = 1.3, *p* = .24). The comparison between ATV-Live and ATV-Anim was also not significant (*t*(1,5) = .33, *p* = .75).

#### Signal-to-noise: static features

**Figure 5:**
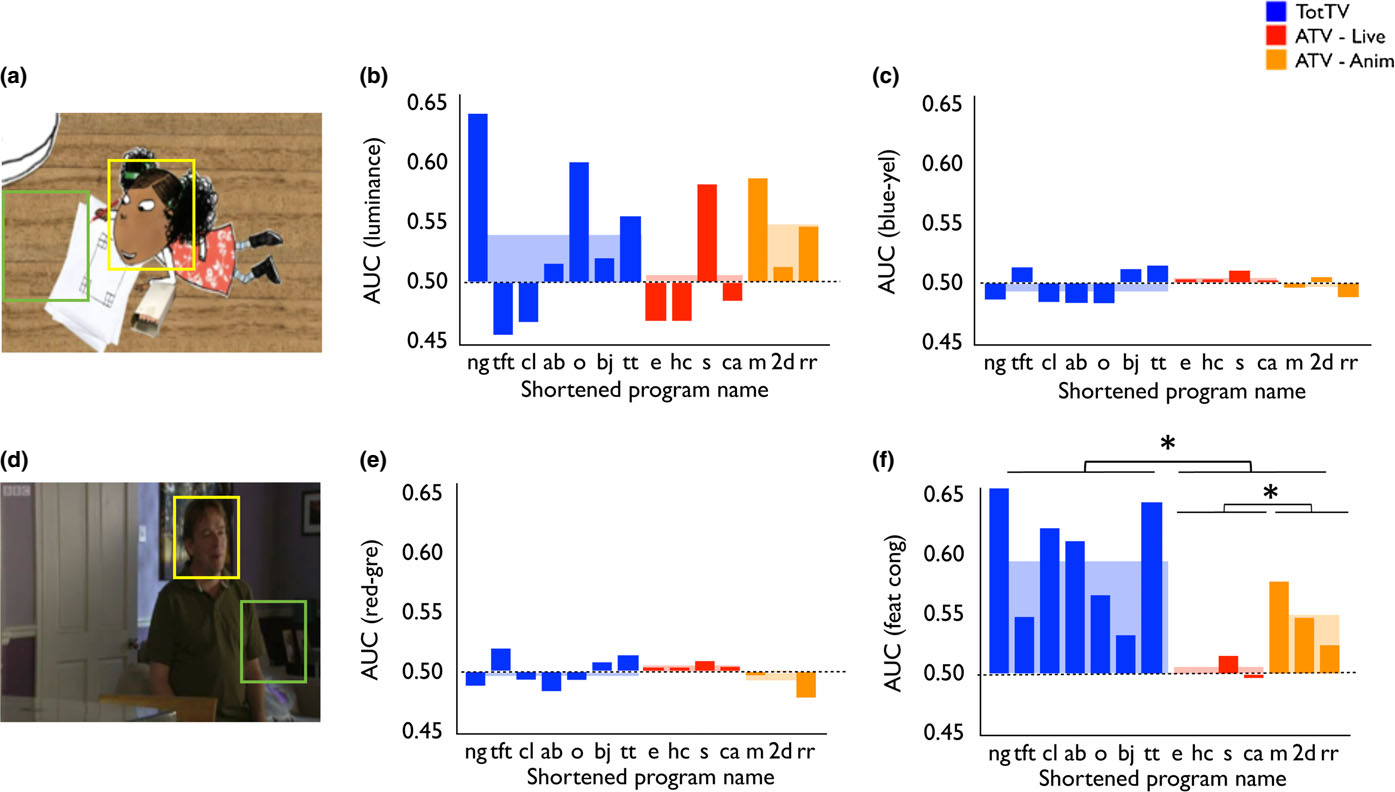
(a) and (d) show sample frames illustrating our ROC analyses. For each frame, the face of the speaking character was identified via hand-coding (marked yellow) and a randomly positioned baseline was generated with the same dimensions (marked green). Higher AUC values indicate that a particular feature dimension was more present in the speaking character face than in the baseline samples, across all the frames that were analysed. (b) AUC for luminance; (c) AUC for blue–yellow intensity; (e) AUC for red–green intensity; (f) AUC for feature congestion. *p < .05.

Next we conducted ROC calculations to assess the degree to which the position of the speaking character was predicted by each of the static feature dimensions (see Figures 5 and S2). Our results are expressed as Area Under the Curve (AUC). A higher AUC indicates that that feature dimension is more predictive of the speaking character location – in other words, that that feature dimension is higher around the face of the speaking character than it is in the rest of the frame.

##### AUC (luminance)

Comparison of TotTV and ATV was not significant (*t*(1,11) = .59, *p* = .59). Comparison of ATV-Live and ATV-Anim was also not significant (*t*(1,5) = 1.2, *p* = .29).

##### AUC (colour intensity)

Blue–yellow dimension: The comparison of TotTV and ATV was not significant (*t*(1,12) = .64, *p* = .54). The comparison of ATV-Live and ATV-Anim was also not significant (*t*(1,5) = 1.6, *p* = .18). Red–green dimension: The comparison of TotTV and ATV was not significant (*t*(1,12) = .15, *p* = .89). The comparison of ATV-Live and ATV-Anim was also not significant (*t*(1,5) = 2.0, *p* = .11).

##### AUC (feature congestion)

AUC was significantly higher in TotTV than ATV (*t*(1,12) = 3.4, *p* < .01), suggesting that feature congestion was significantly more predictive of the location of the speaking character in TotTV than in ATV. AUC was also higher in ATV-Anim than ATV-Live (*t*(1,5) = 3.4, *p* < .05). A direct comparison of ATV-Anim with TotTV was not significant (*t*(1,8) = 1.6, *p* = .16).

In addition to these analyses we also performed a separate calculation addressing the same question: for each feature dimension, we calculated the mean feature values within the speaking character and within the whole frame and compared the proportion of the two. The results of this independent calculation replicated our ROC analyses (see Supplementary Results, Table S1 and Figure S3).

#### Signal-to-noise: dynamic features

**Figure 6:**
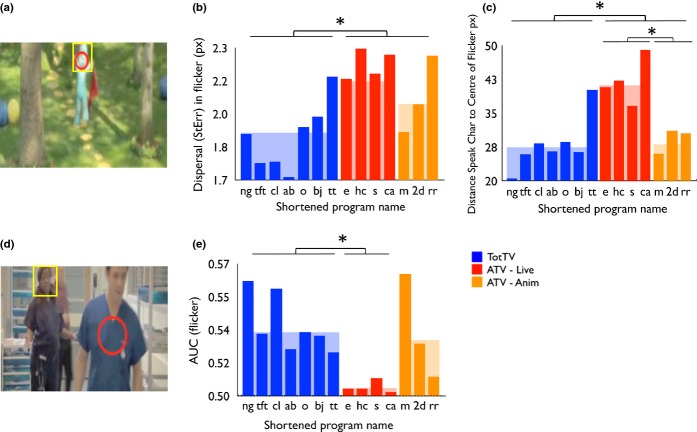
(a) and (d) show sample frames illustrating the flicker analyses (see also Supplementary Videos). In each case the yellow rectangle shows the hand-coded speaking character, and the red circle shows the centre and variance in the flicker, calculated as reported in the text. In (a) the only motion in the frame is the character moving; the position of the centre of the flicker (drawn in red) closely matches the hand-coded position of speaking character's head; (d) shows the speaking character talking to an actor walking through the foreground, with another character walking in the background. The centre of flicker on-screen (drawn red) is some distance from the speaking character. (b) Dispersal in flicker. (c) Distance between the speaking character and the centre of flicker. (e) AUC for flicker, calculated as described in the text and in the legend to Figure 6.*p < .05.

Next we examined movement, approximated via flicker (Figure 6).

##### Average dispersal in flicker

This measure expresses how tightly concentrated the flicker is within a frame – in other words, whether the flicker tends to be spread out across the whole screen or to be concentrated within one particular area. Flicker in TotTV was found to show significantly lower dispersal than ATV (*t*(1,12) = 3.4, *p* < .01). Comparisons of ATV-Live with ATV-Anim (*t*(1,5) = 2.9, *p* = .17) and of TotTV with ATV-Anim (*t*(1,8) = 1.6, *p* = .15) were not significant.

##### AUC (flicker)

The ROC method followed was identical to those used for the static feature dimensions; this calculation examines the degree to which flicker predicts the location of the speaking character face. Comparison of TotTV and ATV was marginally non-significant (*t*(1,12) = 2.0, *p* = .07). Comparison of ATV-Live and ATV-Anim was also marginally non-significant (*t*(1,5) = 2.2, *p* = .08). In addition, we directly compared TotTV and ATV-Live; this was significant (*t*(1,9) = 4.3, *p* < .01).

##### Distance between centre of flicker and centre of speaking character face

We also performed a further calculation to examine the degree to which flicker predicts the location of the speaking character face. Frame by frame, we calculated the distance between the centre of the flicker and the centre of the (hand-coded) speaking character face. On average this was significantly lower in TotTV than ATV (*t*(1,12) = 2.3, *p* < .05). ATV-Live was significantly higher than ATV-Anim (*t*(1,5) = 3.9, *p* < .01) (see also Figure S4). A comparison of TotTV with ATV-Anim was not significant (*t*(1,8) = .32, *p* = .76). This suggests that on-screen flicker is more closely related to the location of the speaking character face in TotTV than in ATV-Live.

## Discussion

This study used corpus analyses to examine the intuitions of TotTV producers. By comparing the level and distribution of low-level visual features we found evidence that the designers of TotTV may have intuited techniques for using low-level features to guide information to the semantically most informative parts of an image.

Our initial analyses suggested that mean shot length was longer in TotTV than ATV (Figure 3), which is surprising given reports that fast-paced editing attracts more attention in infants and young children (Gola & Calvert, 2011; Valkenburg & Vroone, 2004). To our knowledge, no previous research has directly compared editing speeds between TotTV and ATV (although see Goodrich *et al*., 2009; Wright, Huston, Ross, Calvert, Rolandelli, Weeks, Raeissi & Potts, 1984). As with all our findings, future work should investigate whether the infant- and toddler-directed commercial DVDs that have been the focus of other research (e.g. Goodrich *et al*., 2009) show similar patterns to the BBC TV dramas analysed here.

We then examined the degree to which low-level visual features predict the location of the speaking character face. First we found that feature congestion (a combination of differentials of luminance, colour and edge orientation) was significantly more predictive of face location in TotTV than ATV (Figure 5f). There was no difference in the level of feature congestion across the whole frame (Figure 4b). Luminance and colour intensity were, in contrast, not significantly predictive (Figure 5), which is unsurprising because previous research has shown that these features do not predict gaze in dynamic scene viewing (Mital *et al*., 2010). Both these findings were replicated using alternative analyses (see Table S1 and Figure S3).

**Figure 7:**
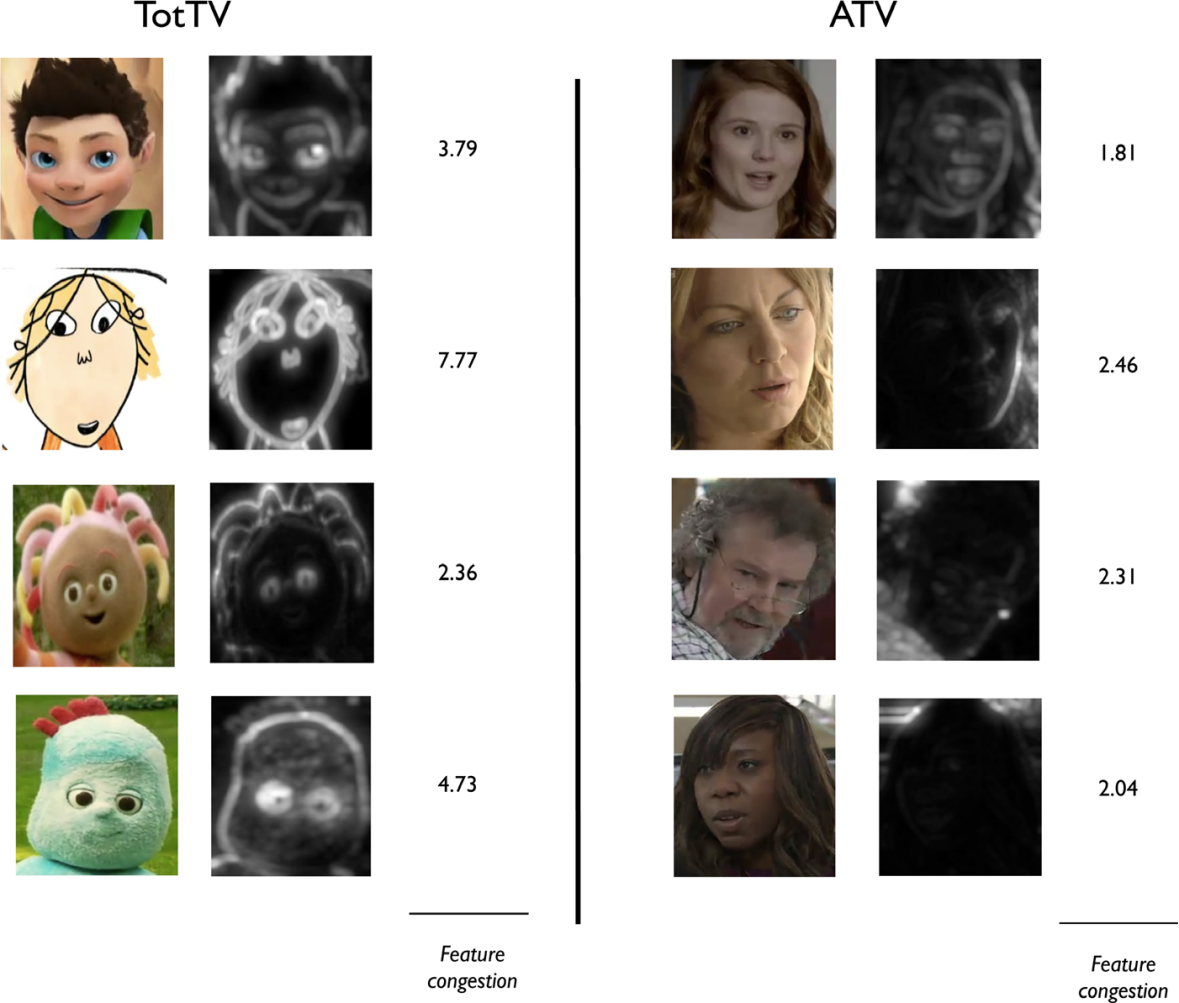
Comparing feature congestion between our TotTV and ATV-Live samples. Almost all of the faces in our TotTV programmes were either CGA, Cel or live action puppets, whereas all the faces in the most popular ATV excerpts were of human actors. For each example, the image on the left shows the input image, identified via hand-coding; the image on the right shows the feature congestion map; light-coloured areas are more congested. The number to the right shows the average feature congestion over the whole face.

Figure 7 shows a sample of character faces analysed for feature congestion. Total feature congestion across the whole face is higher in the TotTV than the ATV samples; also interesting, however, is that in the TotTV samples the areas of highest feature congestion are located around the eyes, which are semantically the most informative areas within the face (Grossman & Johnson, 2007). This was true both for all animated characters (including ATV-Anim as well as TotTV) and for costumed live action TotTV characters. In the ATV-Live samples, in contrast, feature congestion around the eyes does not appear to be higher than it is in the rest of the face (Figure 7) and feature congestion in the face is not higher than in the rest of the frame (Figure 5f). Our findings suggest that feature congestion, which is a low-level exogenous influence on gaze location (Rosenholtz *et al*., 2007), may be used in TotTV to guide attention both to the semantically most important part of the frame (the face) and to the semantically most important part of the face (the eyes). One irony is that their simplified design and limited range of movement means that the eyes of cartoon/puppet characters may not have the full range of nuance and expression that real eyes do. However, for younger viewers this reduced complexity may accentuate critical expressive features and increase comprehensibility, which in turn leads to increased attention (Pempek, Kirkorian, Richards, Anderson, Lund & Stevens, 2010, Kidd, Piantadosi & Aslin, 2012).

The strongest low-level predictor of gaze location is movement (Mital *et al*., 2010). We found that there were no differences in total movement between TotTV and ATV (Figure 4e). We then performed two analyses that appear to suggest that TotTV features compositions in which movement is used more effectively to guide attention to the location of the speaking character within the frame. First we calculated the average distance between the speaking character and the centre of the movement, and found this to be lower in TotTV than ATV (Figure 6c). Second, we used ROC analyses to examine the degree to which the speaking character location was predicted by movement within the frame; we found that this was higher in TotTV than ATV (Figure 6e; see also Supplementary Videos). Both of these findings suggest that TotTV designers are using flicker to guide attention to the speaking character.

We also found that the proportion of the screen occupied by the speaking character face is lower in TotTV than ATV (Figure 3c and 3d). This appears counterintuitive given that visual acuity is lower in young individuals (Mayer & Dobson, 1982) and that faces are semantically informative (Grossmann & Johnson, 2007). However, adult gaze studies have revealed that close shots can lead to greater disagreement between where viewers look on the screen: when a face is close up on the screen it ceases to be attended to as a whole and is instead attended as individual elements (Smith, 2013). The use of long-distance shots in TotTV combined with the greater use of motion contrasts within a shot may assist viewers in finding the semantically important elements in the shot. However, slower infant orienting combined with increased shot size may increase the time needed to encode the shot content. Consistent with this we found a relationship between shot length and shot size in TotTV (see Figure S1) but not in ATV-Live. This relationship has been proposed by film theorists (Bordwell & Thompson, 2010) but to our knowledge has not previously been quantified.

For our findings on both feature congestion and movement, the ATV-Anim group was generally more similar to TotTV than to ATV-Live (Figures 5c, 6e, 6f). This suggests that the differences we identify may be more attributable to differences between animation and live action than to between TotTV and ATV. To our knowledge surprisingly little research hitherto has quantitatively investigated how cartoons differ from live action; our results may give insight into why so many popular TotTV programmes are cartoons, in contrast to ATV in which the most popular programmes are almost exclusively live action. However, it should also be noted that the feature congestion (Figure 5c) and motion differentials (e.g. Figure 6e) we identified were strongest in one of the live action TotTV programmes we examined (Night Garden), suggesting that these differences are not entirely attributable to animation vs. live-action contrasts, but rather may be intentionally imposed on live-action scenarios by TotTV designers. This is perhaps similar to how a clown accentuates facial expressions using bold face make-up.

Our analyses suggest that ATV is structured with high levels of non-essential low-level information content. This may be because adult viewers find it relatively easy to parse out extraneous information (‘noise’) and concentrate on semantically important information (‘signal’). TotTV, in contrast, appears to contain more economical low-level information content structures, in which low-level information is more concentrated around the semantically important parts of the image. These findings complement previous findings suggesting that infant gaze behaviour shows higher entropy than that of older infants and adults while viewing an identical scene (Frank *et al*., 2009; Smith *et al*., under review).

Feature congestion and movement showed no difference on total mean values (Figure 4), suggesting that the differences we identified relate more to the comprehensibility of information (i.e. where within the frame to look) than they do to the simple task of attracting attention to the screen (cf. Gola & Calvert, 2011). Our results may therefore complement previous research suggesting that comprehensibility is important in influencing gaze behaviour during TV viewing (Pempek *et al*., 2010).

In summary, our findings suggest that the designers of TotTV may have intuited techniques for using low-level information content to guide attention to the semantically important elements within the frame. These findings are comparable to research into how adults may have intuited ways of manipulating their speech when talking to infants in order to maximize the comprehensibility of what they say (‘motherese’ – see e.g. Zhang *et al*., 2011). They also speak to other areas of behavioural research that have used eyetracking to investigate how endogenous factors such as semantic relevance play a relatively lower role in guiding gaze allocation in younger individuals (Frank *et al*., 2009; Smith *et al*., under review). Attentional control is weaker in younger individuals (Davidson, Amso, Anderson & Diamond, 2006); low-level information content structures are therefore more important in determining whether materials presented are attended to and understood. Our findings suggest that similar principles may apply to TV design as to speech (Pierrehumbert, 2003) and word learning (Rost & McMurray, 2010), as well as to other areas of learning such as categorization (Grossmann, Gliga, Johnson & Mareschal, 2009).

### Limitations and directions for future work

These findings present a number of avenues for quantifying the comprehensibility of low-level information structures. However, they also contain substantial limitations: first, as presented here they only apply to those frames which contain a speaking character, and second, they are limited to the visual modality. One goal for future work is to extend these analyses so that they also incorporate other aspects of semantically relevant vs. irrelevant information in addition to the face/non-face distinction we have used here, such as when a character uses social cues to direct attention to a semantically relevant object. A second goal, which is of particular interest given the importance of multi-modal cues in guiding attention in young individuals, is that of analysing signal-to-noise ratios within audio as well as visual information (Barr, 2010).

The novel computational techniques presented in this manuscript can also in future be applied to study naturalistic gaze allocation in contexts not involving a screen, using data from a head-mounted eyetracker (Aslin, 2009; Franchak & Adolph, 2011). This will allow us to investigate, within naturalistic contexts, how the relative role that low-level visual cues and content (e.g. face/non-face) play in guiding gaze allocation varies (a) over developmental time, (b) between individuals and (c) contingent on short-term variability in other factors such as arousal (cf. de Barbaro, Chiba & Deak, 2011; Frank, Amso & Johnson, 2014). These techniques also have further potential applications. For example, parents and designers of childhood environments such as toys, playgrounds, and classrooms have intuitively structured the sensory environment to maximize interest, focus attention and aid child learning – but the efficacy of these sensory environments has not to our knowledge been quantified from the child's perspective. Applying the computational analysis of visual features presented in this paper to data from head-mounted eyetrackers will allow for the application of quantitative techniques to address these research questions. The work presented in this paper is a first step toward this objective.

## Footnotes

1 http://www.barb.co.uk/viewing/weekly-top-10?

2 http://www.barb.co.uk/report/weekly-top-programmes-overview

3 http://www.bbc.co.uk/commissioning/briefs/tv/browse-by-genre/cbeebies/

## Supporting Information

**Figure S1.** Scatterplots showing the relationship between shot length and proportion of screen occupied by the speaking character face. Individual datapoints represent individual shots. For each shot the mean character size during that shot has been calculated from all the codable frames for: a) TotTV; b) ATV - Live; c) ATV - Anim. The Pearson product-moment correlations observed between these variables are reported in the main text. **Figure S2.** ROC curves. a) luminance; b) feature congestion; c) blue-yellow intensity; d) red-green intensity; e) flicker. The AUC results have been shown in the main text. **Figure S3.** a) a sample frame illustrating how the analysis was conducted. For each frame, the amount of each feature dimension was calculated independently for the speaking character face and for the rest of the frame excluding the speaking character; the ratio was then calculated. b) luminance; c) feature congestion; d) blue-yellow intensity; e) red-green intensity. **Figure S4.** Frame-by-frame breakdowns of flicker calculations. Separate plots have been drawn for each program. In each case, frame has been drawn on the x-axis. a) shows frame-by-frame values for the dispersal in flicker (shown in Figure 6b). b) shows frame-by-frame values for the distance between the speaking character and the centre of flicker (shown in Figure 6c). **Table S1.** summary results of our analyses looking at the proportion of feature dimensions in speaking character vs in background.
