# Supplementary material for: Visual motherese? Signal-to-noise ratios in toddler-directed television
Source: Dev Sci. 2014 Apr 7;18(1):24–37. doi: 10.1111/desc.12156 (PMC4309493; doi:10.1111/desc.12156)
Supplement: Supplementary file 1 [file desc0018-0024-sd1.docx]

Supplementary Materials

1. Supplementary Methods
   1. Luminance
   2. Feature congestion
   3. Flicker
   4. ROC curves
2. Supplementary Results
   1. Shot length vs proportion of screen occupied by the speaking character
   2. ROC curves
   3. Proportion of feature dimensions in speaking character vs in background
   4. Flicker analyses - frame-by-frame breakdown

1) Supplementary Methods

a) Luminance

We used the 1976 CIE L*a*b* (CIELAB) color-space defined in the ICC profile specification (C.I.E., 1978). CIELAB allows the use of separating color perceptions into 3-dimensional space where the first dimension, L*, specifies Luminance (Lum), and the other two dimensions, a* and b*, specify color opponents for Red-Green (RG) and Blue-Yellow (BY), respectively. When the color channels a* and b* are achromatic, (i.e. both 0), L* extends values along the grayscale. We used the specification implemented in Matlab’s Image Processing Toolbox (see Figure 2). RG and BY intensities were calculated as the distance from the midpoint (grey); higher values indicate more intense colors on the RG and BY spectra.

b) Feature congestion

Feature congestion was calculated using Matlab scripts that are described in detail by Rosenholz and colleagues (Rosenholtz et al. 2007). These were chosen in preference to the more widely used salience models (e.g. Itti & Koch, 2001) since these models make a lot of assumptions about how attention is controlled, such as the inclusion of inhibition of return and winner takes all in the salience computation. We calculated feature congestion instead of salience since we wanted to describe the combination of first-order features in the scenes. Briefly, the input image was converted into the CIELAB color space and then processed at three scales by creating a Gaussian pyramid by alternately smoothing and subsampling the image (Burt & Adelson, 1983). Features were then identified based on luminance contrasts (by filtering the luminance band by a center-surround filter formed from the difference of two Gaussians and squaring the outputs); color, performed at each scale by pooling with a Gaussian filter; and orientation (using a two-vector, (k cos(2θ), k sin(2θ)), at each image location and scale, where θ is the local orientation and k is related to the extent to which there is a single strong orientation at the given scale and location. Then the local (co)variance for each feature is calculated; these are then combined, scaling the clutter value in each feature dimension by the range of possible clutter values for that feature (Rosenholtz et al., 2007).

c) Flicker

Frames in which either the current or the previous frame was a cut frame (as identified using the hand-coding described above) were excluded. For each frame a threshold was identified as those pixels showing flicker >=1% of all pixels in that frame. The locations of those pixels were identified and their centre was calculated. The dispersal was then calculated as the average Euclidean distance of those pixels from the centre of flicker, divided by the number of pixels above threshold. This technique does not allow for the possibility that there are multiple peaks of flicker within a frame that may compete for viewer attention. However, most computational saliency models assume that the “winner-takes-all” when deciding which area of the screen exogenously attracts attention when sampling from a salience landscape with multiple peaks (e.g. Itti & Koch, 2001). In our calculation of flicker dispersal, instances when there are multiple peaks with flicker >=1% of the overall frame flicker will result in a very wide flicker dispersal measure. This is intuitively sound as such moments provide more competition for viewer attention and, therefore a lower signal-to-noise ratio.

d) ROC curves

This analysis has several benefits when investigating feature values, such as an ability to account for the bias of photographers to place salient features near the centre of the screen (Mital et al., 2010). Each feature map within each frame was systematically thresholded from its minimum to maximum value. For each frame a baseline location was generated at a random location with the same dimensions as the hand-identified speaking character. At each threshold, actual and baseline locations were identified as either having mean values above (a “hit”) or below the threshold (a “miss”). If a threshold also labeled a baseline location as a “hit” it constituted a ‘‘false alarm’’. If the systematic thresholding produced as many hits as false alarms, then the feature dimension could not be said to distinguish between the signal (actual locations) and the noise (baseline locations) (Green & Swets, 1966; Mital et al., 2010).

2) Supplementary Results

All supplementary figures included are referred to in the main text.

a) Shot length vs proportion of screen occupied by the speaking character


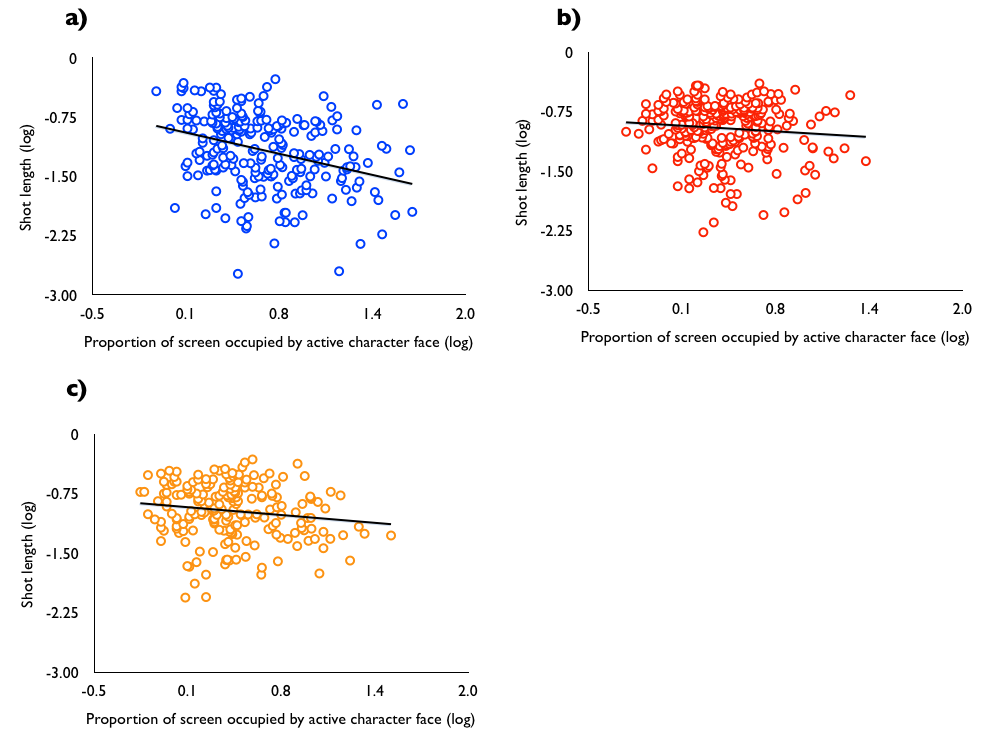


Figure S1 - Scatterplots showing the relationship between shot length and proportion of screen occupied by the speaking character face. Individual datapoints represent individual shots. For each shot the mean character size during that shot has been calculated from all the codable frames for: a) TotTV; b) ATV - Live; c) ATV - Anim. The Pearson product-moment correlations observed between these variables are reported in the main text.

b) ROC curves


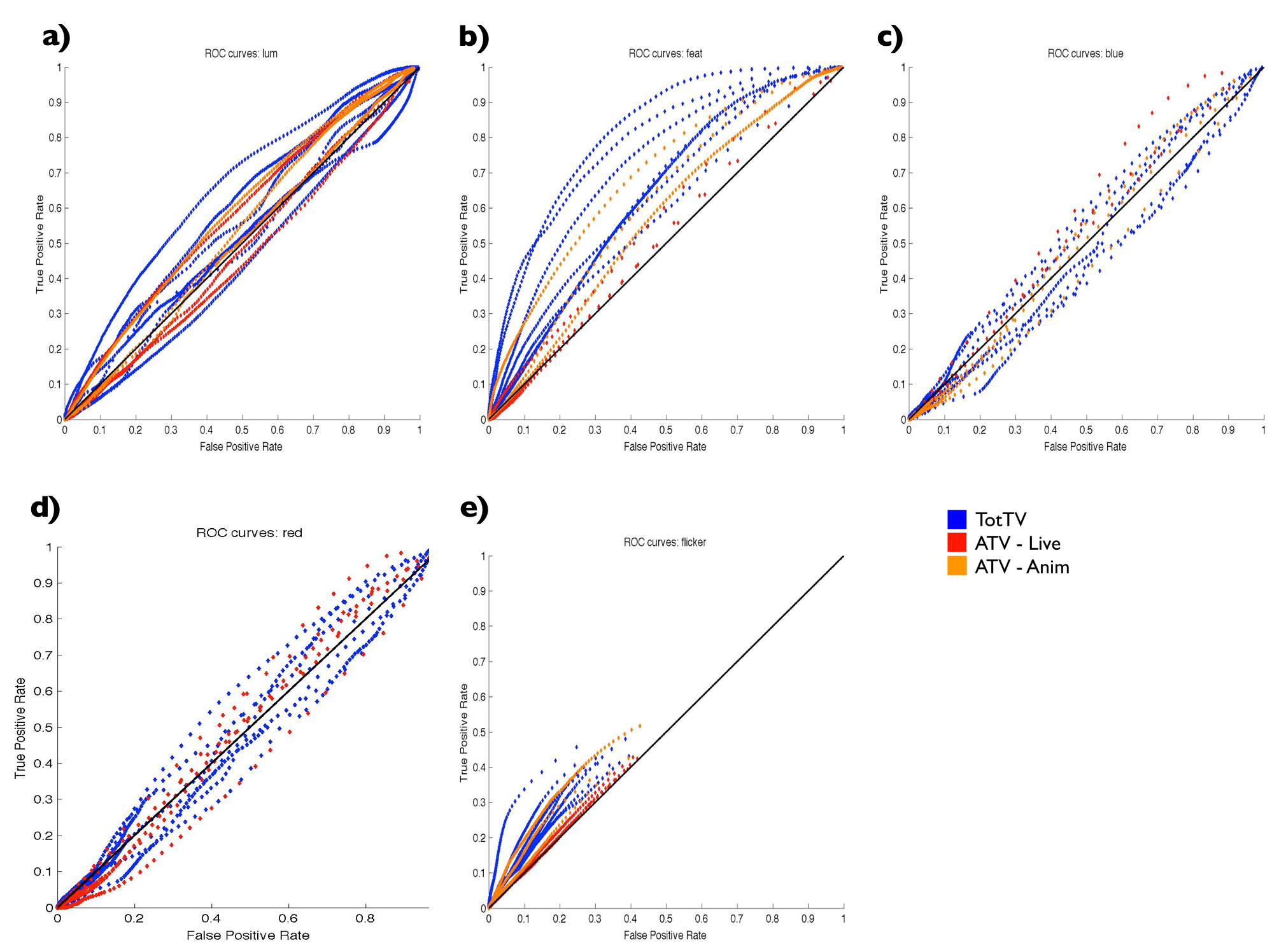


Figure S2 - ROC curves. a) luminance; b) feature congestion; c) blue-yellow intensity; d) red-green intensity; e) flicker. The AUC results have been shown in the main text.

The asymmetry of some of the ROC curves arises from the fact that the feature space was heavily skewed and that linearly spaced thresholds were used (Mital et al., 2010).

c) Proportion of feature dimensions in speaking character vs in background

In order to confirm the validity of our ROC analyses we also conducted a further analysis. For each frame, we calculated the average of each feature dimension within the speaking character face, and the average within the rest of the frame (see Figure S3e). We then calculated the ratio of each feature dimension between the speaking character and the frame; a high value indicates that each feature dimension is more concentrated in the speaking character face than in the frame.

|  | TotTV | ATV | ATV-Live | ATV-Anim | Sig. |
| --- | --- | --- | --- | --- | --- |
| Luminance | 0.26 (0.36) | 0.13 (0.18) | 0.11 (0.22) | 0.16 (0.13) |  |
| Feature congestion | 0.64 (0.23) | 0.18 (0.22) | 0.036 (0.13) | 0.37 (0.16) | TotTV>ATV;  ATV-Anim>ATV-Live |
| Blue-yellow intensity | 0.027 (0.092) | 0.024 (0.017) | 0.030 (0.0056) | 0.014 (0.025) |  |
| Red-green intensity | 0.036 (0.071) | 0.027 (0.0086) | 0.026 (0.011) | 0.028 (0.0072) |  |

Table S1 - summary results of our analyses looking at the proportion of feature dimensions in speaking character vs in background.


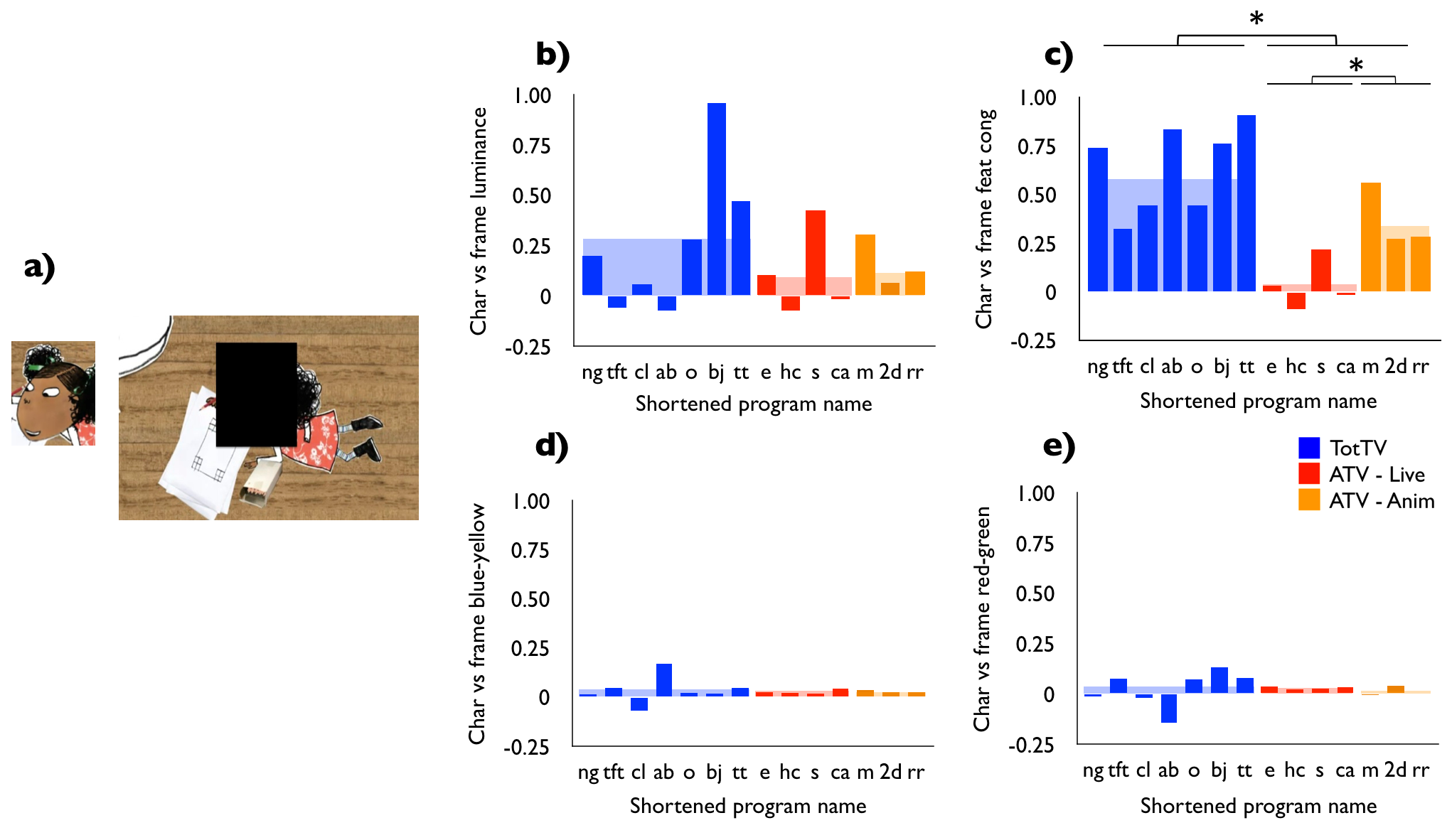


Figure S3 - a) a sample frame illustrating how the analysis was conducted. For each frame, the amount of each feature dimension was calculated independently for the speaking character face and for the rest of the frame excluding the speaking character; the ratio was then calculated. b) luminance; c) feature congestion; d) blue-yellow intensity; e) red-green intensity.

In order to assess the significance of these findings, independent samples t-tests were conducted using an identical analytical strategy to that reported in the main text.

Luminance. The comparison of TotTV and ATV was not significant: t(1,12)=.85, p=.41. The comparison of ATV-Live and ATV-Anim was also not significant: t(1,5)=.37, p=.73.

Feature congestion. The comparison of TotTV and ATV was significant: t(1,12)=3.78, p<.01. The comparison of ATV-Live and ATV-Anim was also significant: t(1,5)=3.0, p<.05.

Blue-yellow intensity. The comparison of TotTV and ATV was not significant: t(1,12)=.09, p=.93. The comparison of ATV-Live and ATV-Anim was also not significant: t(1,5)=1.3, p=.25.

Red-green intensity. The comparison of TotTV and ATV was not significant: t(1,12)=.07, p=.76. The comparison of ATV-Live and ATV-Anim was also not significant: t(1,5)=0.28, p=.79.

These results replicate our findings from the ROC analyses reported in Figure 5.

d) Flicker analyses - frame-by-frame breakdown


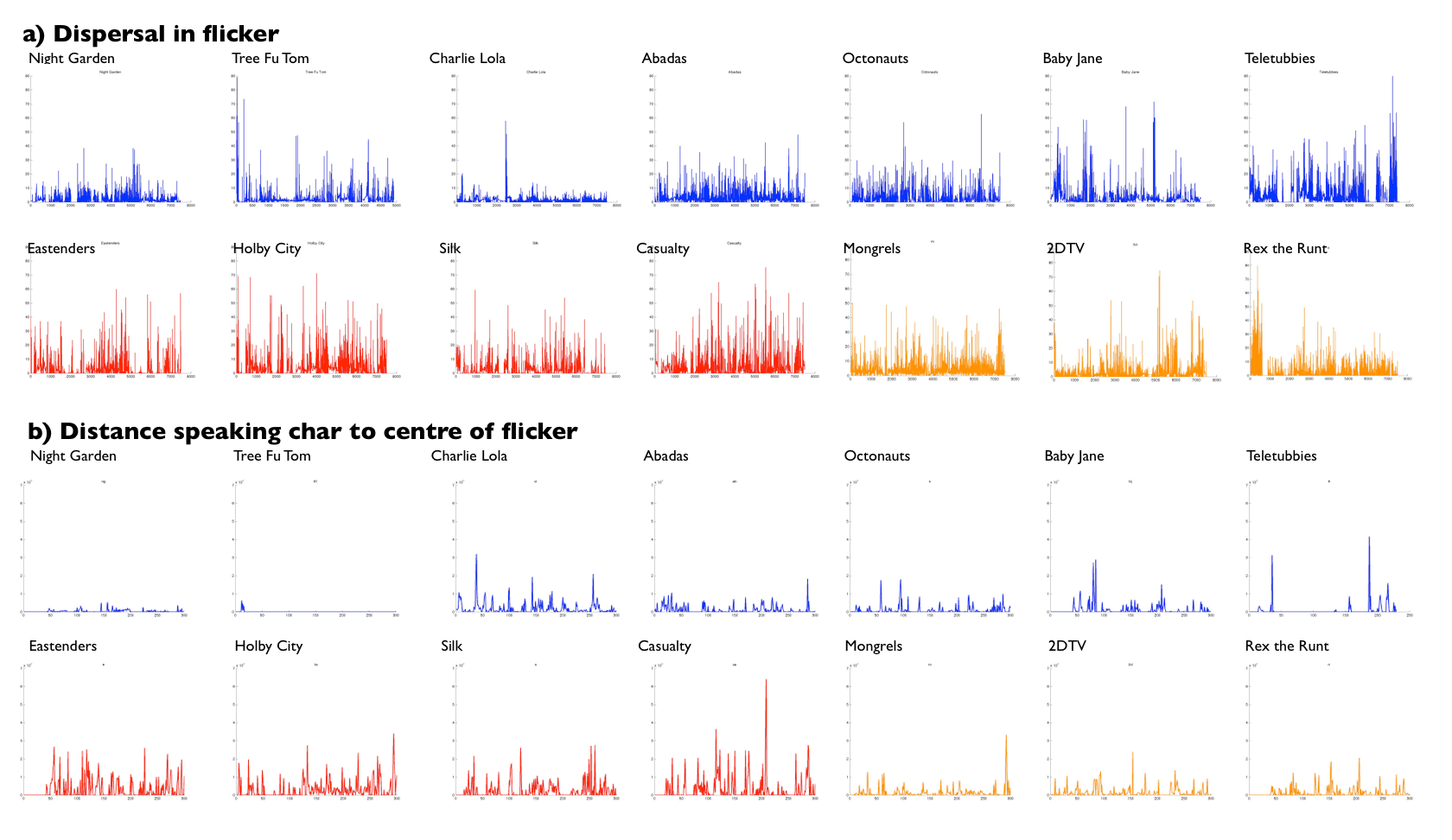


Figure S4 - Frame-by-frame breakdowns of flicker calculations. Separate plots have been drawn for each program. In each case, frame has been drawn on the x-axis. a) shows frame-by-frame values for the dispersal in flicker (shown in Figure 6b). b) shows frame-by-frame values for the distance between the speaking character and the centre of flicker (shown in Figure 6c).
